# Antimicrobial Packaging for Plum Tomatoes Based on ZnO Modified Low-Density Polyethylene

**DOI:** 10.3390/ijms25116073

**Published:** 2024-05-31

**Authors:** Ludmila Motelica, Denisa Ficai, Ovidiu-Cristian Oprea, Roxana-Doina Trusca, Anton Ficai, Maria Daniela Stelescu, Maria Sonmez, Mihaela Nituica, Gabriel Mustatea, Alina Maria Holban

**Affiliations:** 1 Faculty of Chemical Engineering and Biotechnologies, National University of Science and Technology POLITEHNICA Bucharest, 1-7 Gh. Polizu, 011061 Bucharest, Romania; motelica_ludmila@yahoo.com (L.M.); denisa.ficai@upb.ro (D.F.); roxana_doina.trusca@upb.ro (R.-D.T.); anton.ficai@upb.ro (A.F.); alina.m.holban@bio.unibuc.ro (A.M.H.); 2 Academy of Romanian Scientists, 3 Ilfov St., 050044 Bucharest, Romania; 3 National Research and Development Institute for Textile and Leather, Leather and Footwear Institute, 93 Ion Minulescu Street, 031215 Bucharest, Romania; dmstelescu@yahoo.com (M.D.S.); ficaimaria@yahoo.com (M.S.); mihaelavilsan@yahoo.com (M.N.); 4 National R&D Institute for Food Bioresources—IBA Bucharest, Dinu Vintila Street 6, 021102 Bucharest, Romania; gabi.mustatea@bioresurse.ro; 5 Microbiology & Immunology Department, Faculty of Biology, University of Bucharest, 077206 Bucharest, Romania

**Keywords:** polyethylene, LDPE, zinc oxide, *Staphylococcus aureus*, *Escherichia coli*, packaging, tomatoes

## Abstract

Food safety and quality are major concerns in the food industry. Despite numerous studies, polyethylene remains one of the most used materials for packaging due to industry reluctance to invest in new technologies and equipment. Therefore, modifications to the current materials are easier to implement than adopting whole new solutions. Antibacterial activity can be induced in low-density polyethylene films only by adding antimicrobial agents. ZnO nanoparticles are well known for their strong antimicrobial activity, coupled with low toxicity and UV shielding capability. These characteristics recommend ZnO for the food industry. By incorporating such safe and dependable antimicrobial agents in the polyethylene matrix, we have obtained composite films able to inhibit microorganisms’ growth that can be used as packaging materials. Here we report the obtaining of highly homogenous composite films with up to 5% ZnO by a melt mixing process at 150 °C for 10 min. The composite films present good transparency in the visible domain, permitting consumers to visualize the food, but have good UV barrier properties. The composite films exhibit good antimicrobial and antibiofilm activity from the lowest ZnO composition (1%), against both Gram-positive and Gram-negative bacterial strains. The homogenous dispersion of ZnO nanoparticles into the polyethylene matrix was assessed by Fourier transform infrared microscopy and scanning electron microscopy. The optimal mechanical barrier properties were obtained for composition with 3% ZnO. The thermal analysis indicates that the addition of ZnO nanoparticles has increased thermal stability by more than 100 °C. The UV-Vis spectra indicate a low transmittance in the UV domain, lower than 5%, making the films suitable for blocking photo-oxidation processes. The obtained films proved to be efficient packaging films, successfully preserving plum (Rome) tomatoes for up to 14 days.

## 1. Introduction

The majority of food packaging materials used at present are based on petrochemical products or cellulose, due to historical factors like low cost or mechanical and barrier properties [1,2]. The pressure of environmental concerns will phase out petroleum-based materials, which will increase the need for innovative, biodegradable polymeric packaging materials like chitosan [3], alginate [4], cellulose [5], starch [6], pullulan [7], polylactic acid [8], etc. The need to decrease food waste and the desire to increase food safety and prolong shelf life create pressure for the food packaging industry to develop and adopt new antimicrobial materials [9,10]. Unfortunately, despite many research reports, the industry is slow in implementing of novel biopolymers, as novel supply chains and new equipment are needed. One strategy to optimize the costs of novel packaging films is to modify the existing ones by introducing antimicrobial agents that can improve the shelf-life of packed food. This will permit producers to use the existing equipment and add value to their product [11].

As low-density polyethylene (LDPE) does not have antimicrobial activity, introducing antimicrobial agents in the polymer film is the only strategy that can generate polyethylene-based antimicrobial packaging. Various strategies were reported in the literature employing organic [12], inorganic [13], or mixed agents [14]. Inorganic nanoparticles (NPs) represent a special class of antimicrobials that can be used in packaging films; here are worth mentioning ZnO, Ag, CuO, TiO_2,_ or SiO_2_ [15,16,17]. Such innovative antimicrobial biodegradable materials can diminish the microorganisms’ proliferation and thus reduce food spoilage, increase shelf life, and help provide better food quality [18,19,20,21,22]. Among these nanoparticles, silver [23] is the most potent antimicrobial agent, and can bring additional features to a packaging film, like a time–temperature cumulative indicator [24]. Nevertheless, ZnO is considered a safer solution for packaging. Among these nanoparticles, ZnO is considered GRAS (Generally Recognized As Safe) by the US-FDA [25]. The EU recommends a limit of 5–25 mg ZnO/kg of food (article 10/2011 from the Plastics Regulation of the Commission Regulation) [26]. The studies of the European Food Safety Authority (EFSA) concluded that ZnO does not migrate in nanoform and therefore established a specific migration limit (SML) for ionic zinc of maximum 25 mg/kg food [27]. For ionic zinc, no observed adverse effect level was established at 50 mg/day, and an upper limit of 25 mg/person per day was recommended [28]. The scientific literature highlights the antimicrobial activity of ZnO NPs against model pathogens, both Gram- positive or Gram-negative strains [29].

The antimicrobial activity of ZnO is well known, but the exact mechanism is still under debate [30,31]. This is due to the existence of at least three separate pathways: generation of reactive oxygen species (ROS) under light irradiation; mechanical damage generated by nanoparticles, by puncture, rupture, or abrasion, followed by leakage of cellular fluids; internalization of ZnO NPs, followed by the release of Zn^2+^ ions that can bind proteins, lipids, or other essential substances from inside the cell [32].

As ZnO is polar and LDPE is non-polar, the nanoparticles tend to form agglomerates in the ZnO/LDPE composite due to high interfacial tensions. These agglomerates are characterized by: distinct phase morphologies, coalescence, and poor mechanical properties. Two strategies emerged to ensure compatibility: ZnO surface modification with non-polar substances like oleic acid [33] or adding a compatibilizer to the LDPE, like low-density polyethylene grafted with maleic anhydride (LDPE-g-MA) [34,35,36]. The compatibilizer will reduce the interfacial tension and improve the adhesion between the composites’ phases. The ZnO NPs can reinforce the polymer matrix by acting as crosslinking points, generating a stronger material [37], leading to better mechanical properties. The presence of ZnO should also improve the light and gas barrier properties of the packaging [38]. The nanoparticles act as a physical barrier for water or oxygen molecules, decreasing their permeability [39]. At the same time, ZnO is a known sunscreen substance with high UV absorption capacity, which should improve the light barrier properties of any ZnO-based composite.

When it comes to polyethylene-zinc oxide composites, the literature reports focus on either thermal or mechanical properties [40], increasing the homogeneity of the fillers’ dispersion [35], interactions between ZnO and polymer matrix [41], antimicrobial properties [42], and UV shielding [43], but comprehensive reports are missing.

Depositing ZnO on the film surface [44] might enhance the antimicrobial activity, but will lead to a high transfer rate of zinc ions into the food, will not improve the mechanical properties of the film, and can simply fall off due to poor adherence of ZnO NPs to the polyethylene film [45,46,47]. Therefore, including the nanoparticles in the polyethylene matrix will ensure better overall properties, with long-lasting antimicrobial activity, better mechanical and barrier properties, and low migration of the zinc ions into the food [48,49]. Additionally, such composite material will keep its properties when recycled [50], with the ZnO nanoparticles manifesting continuous antimicrobial action. We have used a melt mixing process at 150 °C for 10 min with the help of a Plasti-Corder Brabender mixer, as this will lead to a homogenous dispersion of ZnO nanoparticles into the polyethylene matrix, not only at the surface, improving the mechanical, thermal, and barrier properties of the packaging.

Our aim was to induce antibacterial properties in polyethylene films by adding ZnO NPs by melt mixing, a facile process for packaging manufacturers, and to investigate the influence on the properties of these packaging films. Here we report the influence of ZnO nanoparticles on the mechanical, thermal, structural, and barrier (UV and gases) properties of LDPE films prepared by a melt mixing process at 150 °C for 10 min, with LDPE-g-MA as a compatibilizer, and verify the possibility of using them as antimicrobial packaging films for tomatoes.

## 2. Results and Discussion

### 2.1. UV-Vis Spectroscopy

Transparency is a measure of the ability of a film to block visible light, which protects photosensitive components of food. However, excessive visible light blockage can prevent consumers from visually observing food products. Therefore, a high absorbance in the UV domain coupled with a low one at wavelengths higher than 400 nm is highly desirable.

**Figure 1:**
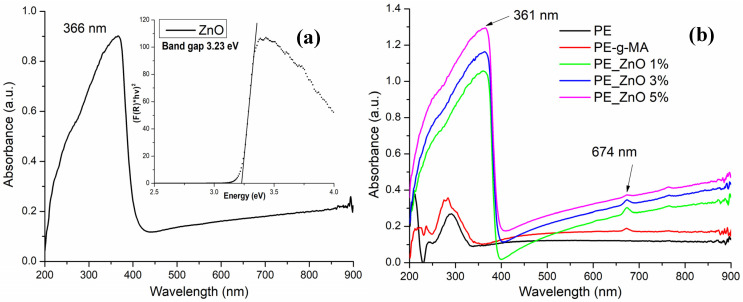
The UV-Vis spectra for (**a**) ZnO; (**b**) PE, PE-g-MA, PE_ZnO 1%, PE_ZnO 3%, and PE_ZnO 5% films.

While the LDPE is transparent, the ZnO is white, therefore presenting little absorption in the visible domain (400–800 nm); nevertheless, the ZnO NPs exhibit a strong absorption band in the UV domain, with the peak at 366 nm (Figure 1).

This UV-centred absorption band is responsible for the ZnO used as sunscreen in cosmetics or as a protective coating, for example, in the textile industry. The fundamental mechanism by which this absorption peak appears is related to the electron jump from the valence band to the conduction band [51], and it has no relation to surface plasmon resonance (SPR), which is specific to metallic particles [52]. Unfortunately, the literature abounds with such mistakes [53,54] and if allowed to become rooted, they will become difficult to combat along with other misconceptions [51]. Some of the most obvious mistakes are when ZnO is considered a metallic particle [55,56] to claim a SPR. As many researchers report the synthesis of Au, Ag, and ZnO NPs by using plant extracts, some misunderstandings are gaining traction when the proposed mechanism for metallic NPs formation and their SPR are applied indiscriminately to ZnO NPs [57]. Therefore, along the non-existent SPR of ZnO, one can read about the bioreduction of Zn^2+^ ions to ZnO, which calls for elementary notions as oxidation states [58].

The peak at 674 nm is due to the presence of the compatibilizer (LDPE-g-MA), as can be seen by comparing the PE and PE-g-MA spectra in Figure 1. As the mass percent of LDPE-g-MA in composite samples is constant, it can be seen that the peak at 674 nm is decreasing in intensity as the ZnO percent is increasing. This can be due to the interactions between compatibilizers’ polar moieties and ZnO NPs. The same interactions are responsible for the hypsochromic shift of the ZnO peak to 361 nm.

**Figure 2:**
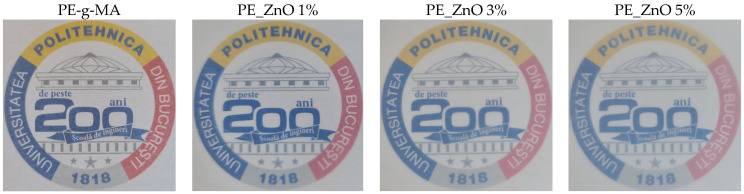
The transparency for PE-g-MA, PE_ZnO 1%, PE_ZnO 3%, and PE_ZnO 5% films.

The composite samples present a low absorption in the visible domain, coupled with a strong peak in the UV region. As such, the main optical request for a packaging film is fulfilled. The films are rather transparent under visible light (Figure 2), permitting customers to see the packed food while at the same time blocking the high-energy photons that are responsible for the initiation of oxidation reactions in lipids, vitamins, and other less stable nutrients.

A higher opacity value indicates that the film is less transparent (Table 1). As expected, the composite films present a higher opacity when compared to the simple polyethylene sample, but there is not much difference among the samples as the percentage of ZnO NPs increases from 1 to 5%. A film with opacity <2 can be considered transparent [59].

### 2.2. FT-IR Spectroscopy and Microscopy

**Figure 3:**
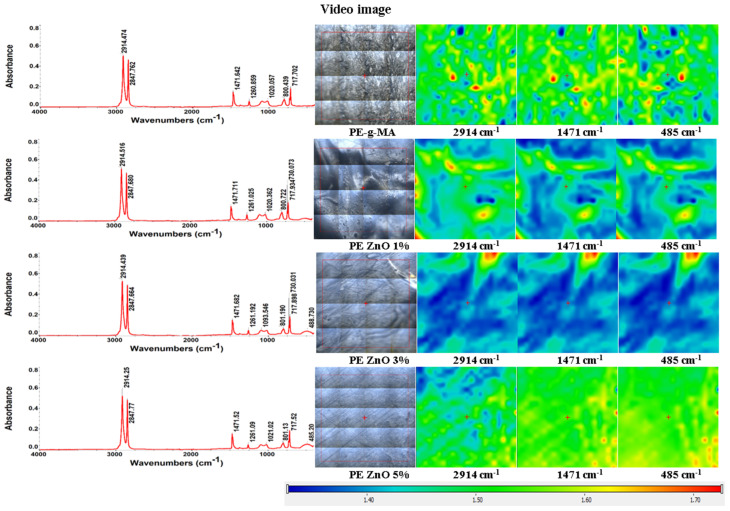
FTIR spectra and microscopy maps of polyethylene/ZnO composite films at 2914, 1471, and 485 cm^–1^; red areas indicate the highest absorbance, while blue areas correspond to the lowest absorbance.

The FTIR spectra of polyethylene and composite samples (Figure 3) present two strong and sharp absorption bands at 2914 and 2848 cm^−1^, assigned to the asymmetric and symmetric stretching vibrations of the -CH_2_- group, specific to the LDPE.

**Figure 4:**
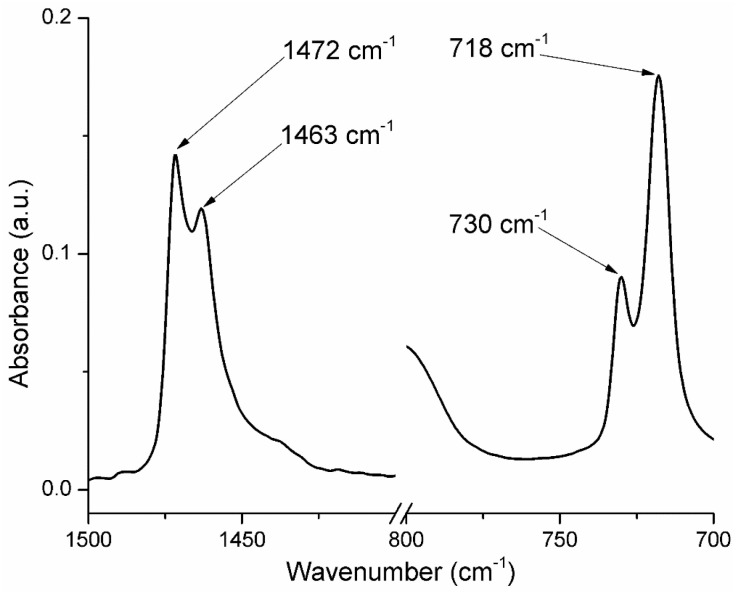
Detail of FTIR spectra of the PE ZnO 5% sample for the wavenumber intervals 700–800 cm^−1^ and 1400–1500 cm^−1^.

At 717–718 cm^−1^ a sharp peak, with medium intensity can be observed, which is attributed to the in-plane deformation vibration (rocking) of the methylene group -CH_2_- [60,61,62]. The small split-off peak from 729 to 730 cm^−1^ is called crystal splitting (Figure 4).

A similar splitting can be observed at 1463–1472 cm^−1^ (CH_2_ bending deformation), with the presence of LDPE-g-MA and ZnO NPs acting as nucleating agents that led to a short-range ordering of the samples [39]. Among these bands, those at 1463 and 718 cm^−1^ refer to the amorphous phase, and those at 1472 cm^−1^ and 730 cm^−1^ are generated by the crystalline phase [63,64,65]. The samples containing ZnO nanoparticles exhibit an absorption band at 484–488 cm^−1^ which is assigned to Zn-O vibration. The principal peaks and their assignments are presented in Table 2.

The FTIR microscopy images (Figure 3) present the spatial distribution of ZnO within the polyethylene composite. Figure 3 illustrates the FTIR 2D maps corresponding to the obtained films at the specific wavenumbers of 2934 cm^−1^, 1480 cm^−1^ and 685 cm^−1^, along with the microscopic view of the subjected region. Red areas indicate the highest absorbance, while blue areas correspond to the lowest absorbance.

The recorded FTIR 2D maps indicate a good homogenization of the ZnO NPs with the simple and functionalized polyethylene, favoring a good dispersion of the inorganic nanoparticles into the final composite. As it can be observed from Figure 3, the samples are quite homogenous, with only minor differences being present at the level of tens of µm maximum. The presence of the compatibilizer, LDPE-g-MA, reduces the agglomeration tendency and improves the dispersion of the ZnO NPs into the LDPE matrix.

### 2.3. Photoluminescence Spectroscopy

Changes in the fluorescence of materials are usually related to surface modification and the presence of additional interactions. Therefore, along with FTIR spectroscopy, this technique is a valuable instrument to understand the existence of surface interactions between ZnO NPs and polyethylene matrix.

**Figure 5:**
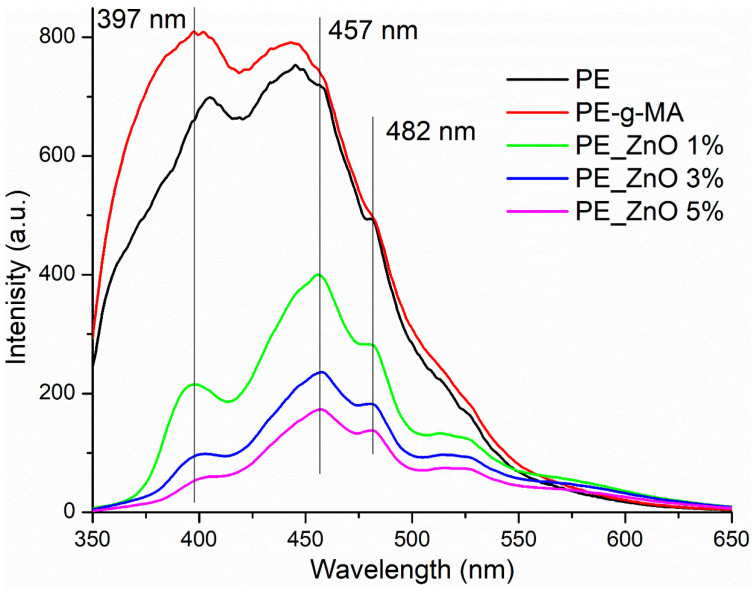
The fluorescence spectra for PE, PE-g-MA and PE_ZnO 1–5% films.

The photoluminescence (fluorescence) spectra of the composite films are presented in Figure 5.

Both PE and PE-g-MA present a high fluorescent emission in the UV and violet-green domains of the visible spectra. The introduction of ZnO NPs is decreasing the intensity of emission of composite films (to 25–50% of the initial visible intensity and to 6–25% for the UV band); the higher the ZnO percent, the lower the fluorescence emission becomes. Additionally, minor shifts of some peaks are recorded, e.g., the 444 nm maximum is bathochromic shifted to 457 nm.

The shape of the emission spectra for composite samples is typical for the presence of ZnO NPs [66], with two specific regions. The emission band in the UV region, called near-band edge emission (NBE), is generated by free exciton recombination. When it has a lower intensity than the emission in the visible domain, it can be considered that the ZnO NPs have a high surface defect density, which generates active centres for fluorescence, photocatalytic activity, and also antimicrobial activity. The presence of these surface defects can act as physical traps for free electrons, which will block the recombination and therefore decrease the NBE emission intensity [67,68,69]. Nevertheless, the intensity of fluorescence spectra for ZnO containing composites is much lower than previously reported for free ZnO NPs, indicating the presence of strong surface interactions between nanoparticles and the polymer matrix [70,71].

### 2.4. Thermal Analysis TG-DSC

**Figure 6:**
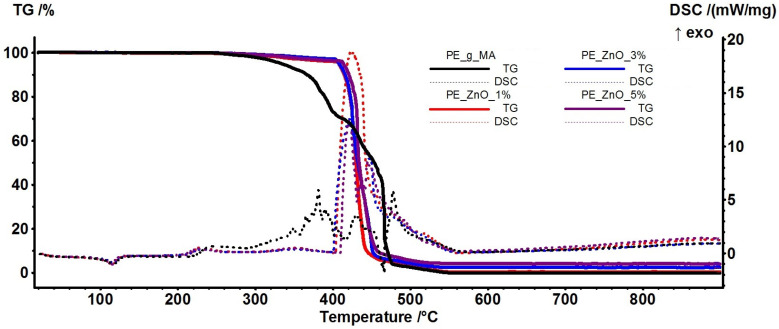
TG-DSC curves for PE-g-MA and PE_ZnO 1%, PE_ZnO 3%, and PE_ZnO 5% composite films.

The thermal analysis results are presented in Figure 6. As it can be observed, the thermal stability of the composite films is higher than the control one, with the introduction of ZnO NPs as fillers increasing the stability of polymer chains. The ZnO NPs act as crosslinking points for the polymer chains, stabilizing them and dissipating excess energy. Therefore, the thermal stability is enhanced by ~100 °C, up to 400 °C, when compared with the control film. Similar findings were reported in the literature [13,33,72], but with a lower stabilization effect from the ZnO NPs. The presence of ZnO NPs in the final composite is improving the long-term stability of the material (ageing) as it can generate ROS that can act as scavengers for LDPE fragments, blocking further the propagation of radical reactions and, therefore, slowing degradation.

The principal numeric data are presented in Table 3, together with the calculated crystallinity degree as a ratio between measured melting enthalpy (ΔH_melting_) and the melting enthalpy of 100% crystalline polymer reported by Wunderlich (293 J/g) [73,74].

The melting process starts at ~106 °C for the composite samples, about 2 °C lower than the control sample. The mass loss up to 300 °C is ~1% for composite samples and double, ~2% for the control film, mostly originating in the partial oxidation of small-molecule plasticizer. The composite samples further lose 2–3% of their initial mass between 300 and 400 °C, while the control film exhibits a mass loss of ~25% in the same interval. This proves the remarkable thermal stability induced by the presence of ZnO NPs dispersed among polymer chains (Figures S1 and S2).

The calculated crystallinity degree is decreasing, especially for the 3 and 5% ZnO samples. This can be explained by considering the dispersion of ZnO NPs, which act as crosslinking points for LDPE-g-MA and alter the ordered pattern of polymer chains.

**Figure 7:**
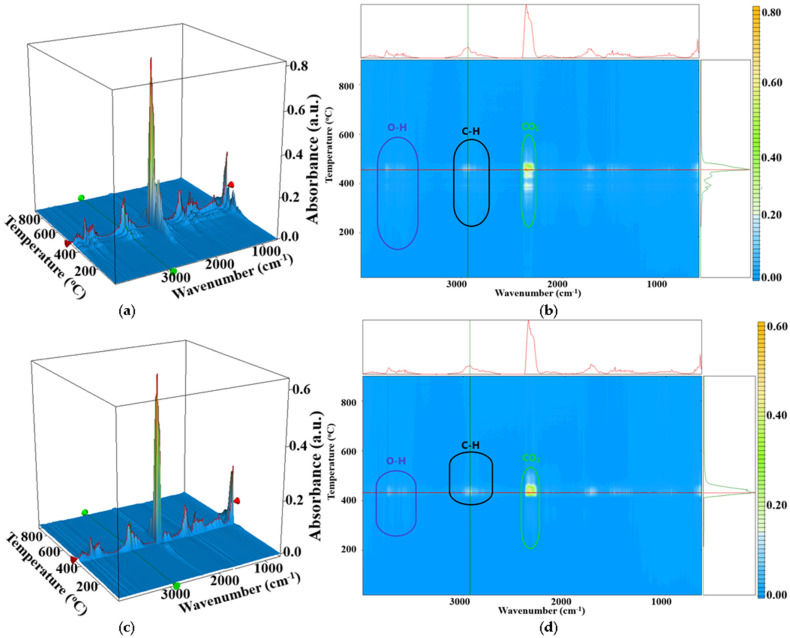
The FTIR 3D diagram for the PE_g_MA–control (**a**) and PE_ZnO 5% (**c**), respectively, their 2D projections in the temperature/wavenumber plane PE_g_MA–control (**b**) and PE_ZnO 5% (**d**); On top of each 2D projection is the FTIR spectrum at the temperature of the highest decomposition rate (464 and 430 °C); on the right side of each 2D projection is the evolving trace for the wavenumber 2934 cm^−1^ assigned to the C-H asymmetric vibration from –CH_2_ moieties.

The FTIR analysis of evolved gases (Figure 7) indicates the presence of a large quantity of CO_2_ (2354 cm^−1^) and some H_2_O (3500–3900 cm^−1^) or CO (2100–2200 cm^−1^) resulted from oxidation reactions, but also an important amount of hydrocarbon fragments resulted from the polymer backbone fragmentation (wavenumbers around 3000 cm^−1^). The enhanced thermal stability can also be observed from the temperature interval where hydrocarbon fragments start to be identified. For the control sample, all the evolved gases, H_2_O, CO_2_, CO, or C-H fragments are being eliminated at lower temperatures when compared with the PE ZnO 5% sample.

### 2.5. Scanning Electron Microscopy (SEM) Characterization

**Figure 8:**
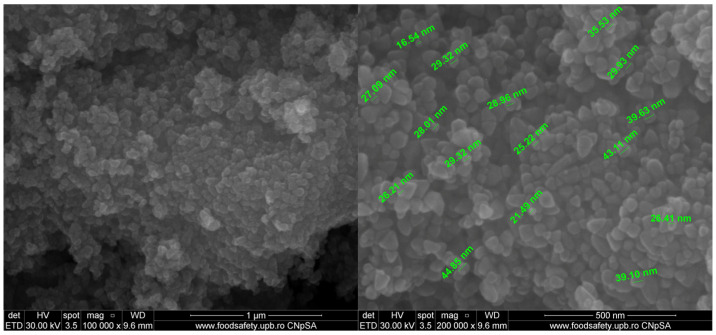
The SEM micrographs for the ZnO nanoparticles.

The SEM micrographs for ZnO NPs are presented in Figure 8. The shape of ZnO NPs is polyhedral, with a uniform size and low agglomeration tendency.

**Figure 9:**
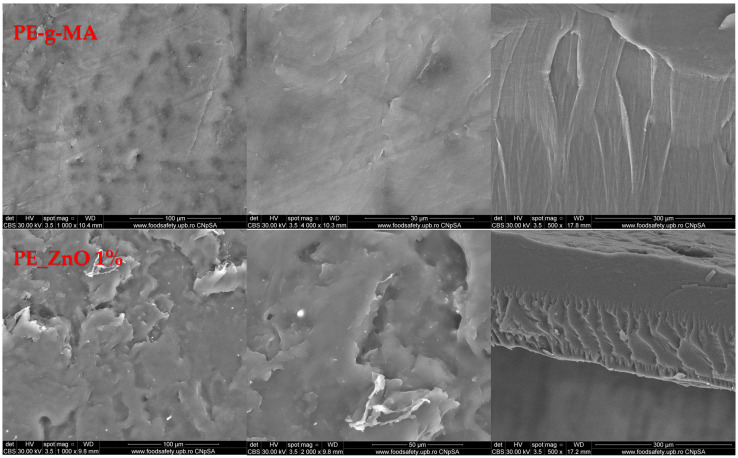

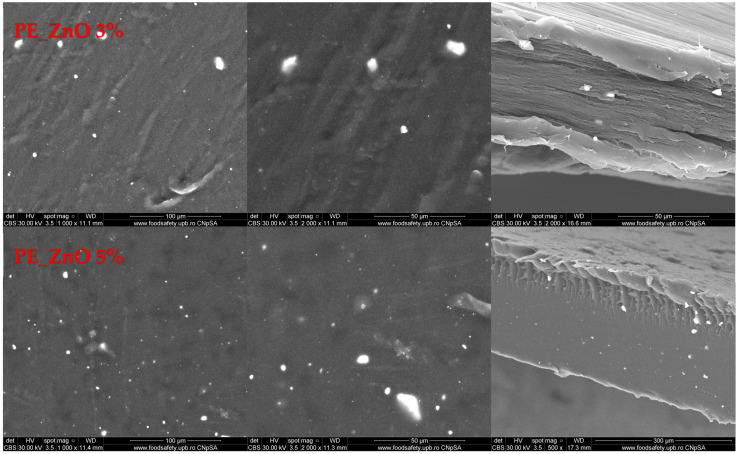
The SEM micrographs for PE-g-MA and PE_ZnO 1%, PE_ZnO 3% and PE_ZnO 5% composite films.

The SEM analysis for the polymer-based samples gives relevant data about surface morphology and other defects that might occur inside or on the surface of the films. The morphology of the composite films is presented in Figure 9. The surface can be considered smooth, with some occasionally scale-like structures for the PE-g-MA and PE_ZnO 1% samples. At higher magnification, the SEM micrographs indicate that on the surface of the films there are random small ZnO agglomerates, visible as white dots. The cross-section micrographs indicate that the composite films are compact, without visible porosity, with a lamellar structure and ZnO NPs well dispersed into the thickness of the films.

**Figure 10:**
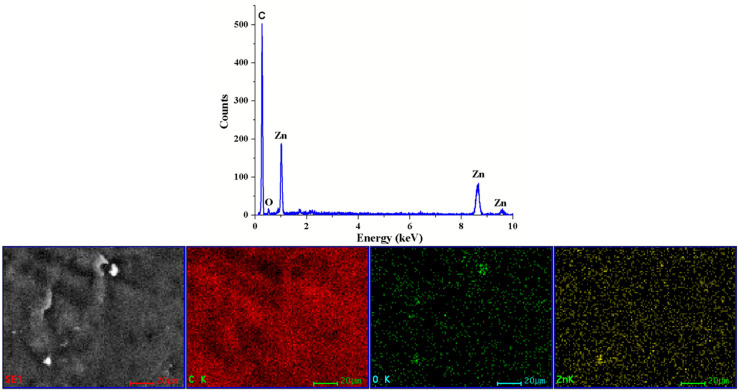
The EDS determined the elemental composition and elemental distribution maps for the PE_ZnO 5% composite film.

The energy dispersive spectrum analysis (EDS) can provide a sharp picture of the homogeneity degree (Figure 10). The elemental maps indicate the rare existence of very small agglomerates of ZnO NPs. Moreover, some defects observed in SEM micrographs can be assigned to matrix imperfections rather than to ZnO agglomerates. The distribution of each element (C, O, and Zn) was determined with the help of the corresponding Kα line from the X-ray spectra. The elemental map images confirm the homogeneity of the films, as indicated by the FTIR maps as well.

### 2.6. Mechanical and Barrier Properties

Films must prevent the transfer of moisture from the external environment to the food during storage, which affects the shelf life of the food; therefore, composite films must have low water vapor permeability (WVP) values. However, the excessive addition of ZnO nanoparticles might result in their presence as free particles in the composite, with fewer interactions mediated by the compatibilizer molecules, leading to the formation of discontinuous microstructures, which can reduce the mechanical properties of the films.

The mechanical properties of the composite films are presented in Table 4. The influence of ZnO NPs leads to an increase in tensile strength and then a decrease in the final composition. The tensile strength increased from 15.2 MPa for PE-g-MA to 18.9 MPa for PE_ZnO 3%, and then was reduced to 15.5 MPa for the 5% ZnO NPs composite.

The elongation at the break has a non-uniform variation. For low amounts of ZnO NPs, the value increases up to 46.1% for 3% ZnO, as the nanoparticles act as crosslinking points among the polymer chains, allowing them to move independently but still remaining in one structure. Adding more ZnO NPs (5%) leads to a sharp decrease in elongation at the break value (14.6%). This is most probably due to multiple bonding points that are stiffening the structure and do not permit the free movement of polymer chains, as the SEM micrographs did not identify discontinuous microstructures that can act as failure zones.

Ideally, through packaging films for food, the substance exchange with the environment should be negligible (loss of water, flavor, and other specific substances) [75]. Therefore, WVP values are very important, as WPV controls the moisture migration between food and the outside of packaging. In general, the microbial spoilage of food can also be associated with a high value for WVP.

**Figure 11:**
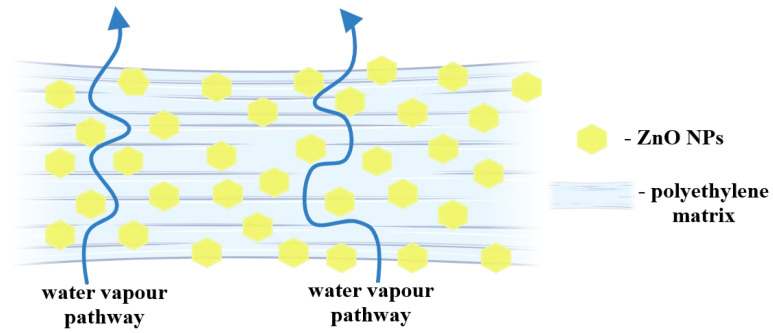
Schematic depiction of the tortuous pathway followed by water molecules passing through the composite films.

The introduction of ZnO NPs between polymer chains can lead to a larger distance among backbones and therefore create spaces for water molecules to diffuse through composite film. On the other hand, the hydrophobic nature of ZnO is diminishing the capacity of water vapors to penetrate the films. The nanoparticles are acting as a physical barrier, increasing the pathway for water molecules and generating a more tortuous route (Figure 11). The combination of these two effects is what generates the end results. For all composite samples, pathway blocking was the predominant effect, with the permeability decreasing, for both O_2_ and H_2_O vapors (Table 4).

### 2.7. Antimicrobial Activity Assay

The evaluation of the growth inhibition results (Table 5) indicates that the presence of ZnO NPs bestows antibacterial properties on the composite films, with the control PE film exhibiting no antibacterial activity.

Although the diameter of the growth inhibition zone increased as the ZnO NPs concentration varied from 1 to 5%, the values were not statistically different. Overall, *E. coli* is slightly more susceptible than *S. aureus* to the presence of ZnO NPs; similar reports have been found in the literature [9,66].

**Figure 12:**
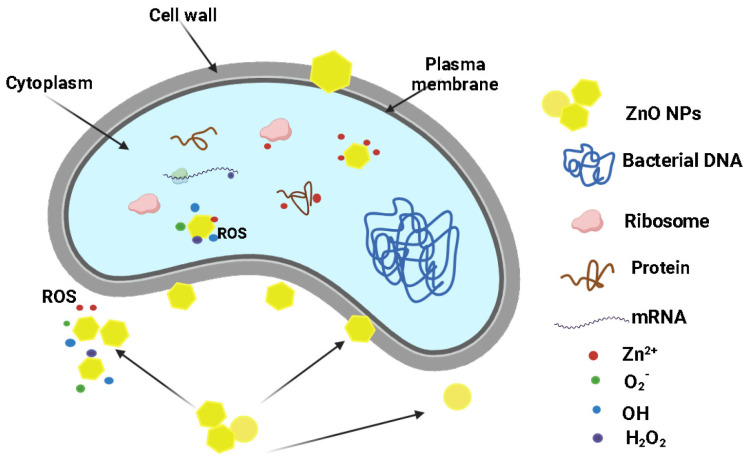
Schematic depiction of the ZnO NPs antibacterial mechanisms.

As polyethylene has no antibacterial activity, the results are generated by the presence of ZnO NPs. Overall, the literature reports three distinct mechanisms for the antimicrobial activity of ZnO (Figure 12).

As a bacterial cell approaches the surface of the ZnO NPs, it encounters a zone where ROS production is taking place, and therefore the concentration of ROS is at its maximum. Further damage to the cellular membranes can occur due to oxidation processes. The oxidative stress generated by the accumulation of ROS is the main bactericidal mechanism of the ZnO NPs [76]. A second mechanism implies mechanical damage to the plasma membrane, by puncturing or rupture. This process leads to the leakage of cytoplasmic components (proteins, nucleic acids, etc.) [76,77]. Responsible for such activity are the ZnO NPs that are embedded at the film surface. Finally, the third mechanism is related to the penetration of the cellular membrane and the internalization of ZnO NPs. ROS production can be resumed inside the microbial cell with more devastating effects. Therefore, the capacity to penetrate the bacterial membrane promotes the nanoparticle to a higher efficiency state, where ROS are delivered inside the cell. Additionally, Zn^2+^ ions are released from the nanoparticles surface and contribute to cell death through cytotoxicity by binding essential components and nutrients of the cell [78].

**Figure 13:**
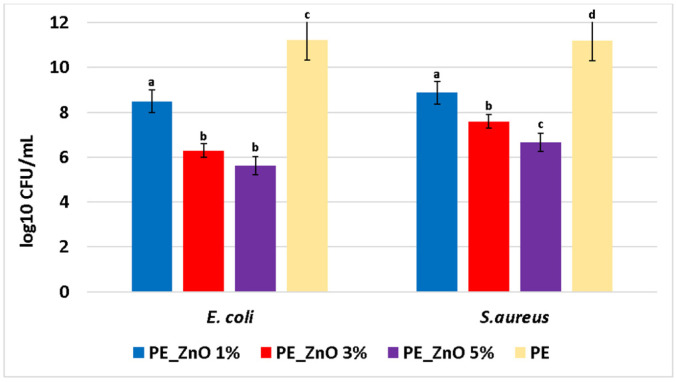
Graphical representation of log_10_ CFU/mL values obtained for the tested *E.coli* and *S. aureus*, expressing biofilm embedded cells developed on PE_ZnO 1%, PE_ZnO 3%, PE_ZnO 5%, and control (PE) composite films after 24 h incubation. Different small letters indicate statistically significant differences between films (*p* < 0.05).

An important property of antimicrobial packaging films is their capacity to combat biofilm formation on their surface. Therefore, the composite films were tested for surface biofilm development (Figure 13). In this case, the attachment of planktonic cells and biofilm development are significantly reduced after 24 h of incubation, indicating a direct correlation with the ZnO concentration. The highest biofilm inhibition was observed for the sample PE_ZnO at 5% for both bacterial strains. At the same time, the effect is stronger against Gram-negative *E. coli* when compared with Gram-positive *S. aureus*.

**Figure 14:**
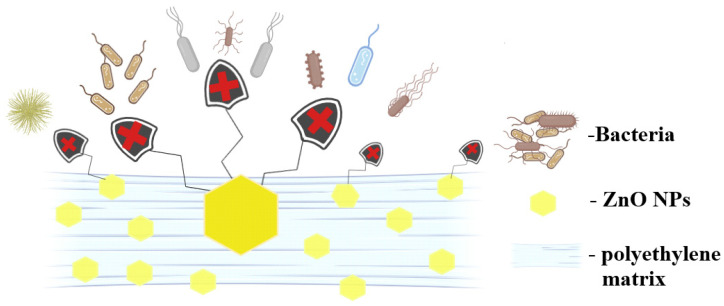
Antibiofilm activity of ZnO NPs at the surface of the polyethylene-ZnO composite films.

Understanding microbial adhesion and retention is crucial for controlling many processes, including biofilm formation. Disrupting the microbial ability to adhere to the packaging materials can prevent further contamination of food [79]. For the polyethylene–zinc oxide composites, the presence of the ZnO NPs at the film surface impairs the bacterial colonization of the samples, blocking the biofilm formation as depicted in Figure 14.

The most likely mechanisms involve the presence of nanoparticles embedded onto the film surface that are producing ROS and can also mechanically interact with the bacterial membrane. Zinc ions released from the nanoparticle surface might also play an important role in blocking bacterial proliferation.

### 2.8. Preliminary Tests on Preserving Tomatoes

Microbiological contamination of food can shorten shelf life, and the presence of antibacterial ZnO NPs in food packaging can slowly act to prevent or kill microorganisms. At the same time, the composite films have better barrier properties, blocking water loss from the packed food.

Oval-shaped tomatoes were packed with the obtained composite films and stored at 4 °C ± 1 °C and 75% relative humidity (RH) for two weeks. A control lot was wrapped in simple polyethylene film.

**Figure 15:**
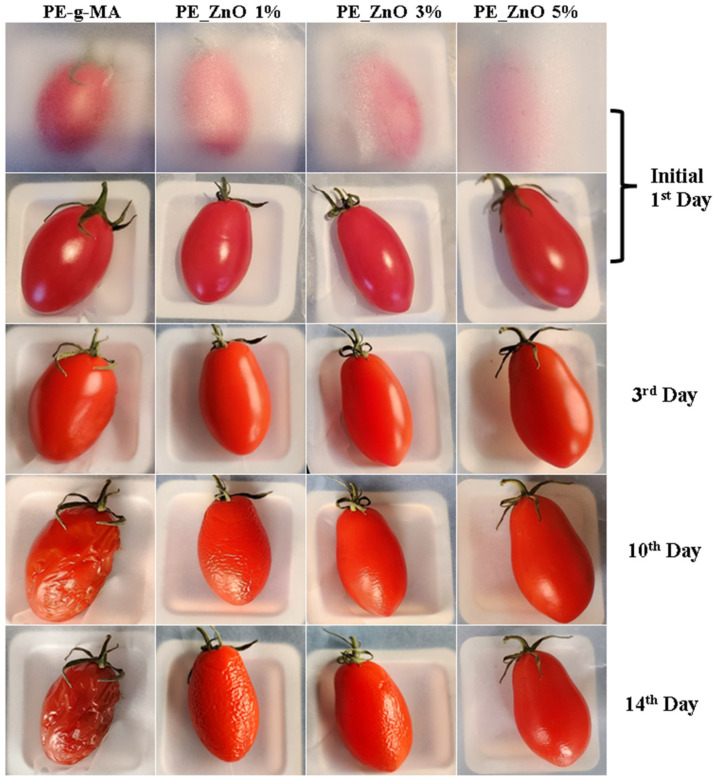
The visual appearance of plum tomatoes stored for 14 days in the polyethylene based packaging films.

The preliminary visual quality check (Figure 15) and weight loss data (Table 6) indicate that polyethylene/ZnO composite films were effective in preserving the tomatoes. The samples wrapped in composite films were clearly different from the control sample, with the tomatoes preserving better the shape, color, and consistency. The weight loss increases with storage time, but there are important differences among samples; the composite film with the highest ZnO content preserves the tomatoes better.

## 3. Experimental Section

### 3.1. Materials and Methods

#### 3.1.1. Materials

Zinc acetate dihydrate (Zn(CH_3_COO)_2_∙2H_2_O) with 99.9% purity and absolute ethanol and n-butanol were acquired from Merck (Redox Lab Supplies, Bucharest, Romania). Low-density polyethylene (LDPE) TIPOLEN MF 243-51 was obtained from MOL Petrochemicals Co., Ltd., Tiszaújváros, Hungary, while polyethylene grafted with maleic anhydride (LDPE-g-MA) Admer NF 468E was provided by Mitsui Chemicals Europe GmbH, Düsseldorf, Germany.

#### 3.1.2. Instrumental Analysis

A STA 449 C F3 piece of equipment from Netzsch (Netzsch, Selb, Germany) was used for the thermogravimetric analysis coupled with differential scanning calorimetry (TG/DSC). The samples (~20 mg of each film) were placed in an open Al_2_O_3_ crucible and heated up with a 10 °C∙min^−1^ rate until 900 °C in a dynamic atmosphere (flow of 50 mL∙min^−1^ of dried air—20% O_2_ and 80% N_2_). An empty alumina crucible was used as a reference. The evolved gases were transferred through heated transfer lines and analyzed on the fly with the help of a FTIR Tensor 27 from Bruker (Bruker Co., Ettlingen, Germany), equipped with an internal thermostatic gas cell. Crystallinity % was calculated as indicated by Equation (1):

*crystallinity* (%) = ΔH_sample melting_/ΔH_100% crystalline melting_ (1)

A Nicolet iS50R spectrometer (Thermo Fisher Scientific, Waltham, MA, USA) was used to record the Fourier transform infrared spectra (FTIR). All measurements were performed at room temperature using the attenuated total reflection (ATR) accessory (Thermo Fisher Scientific, Waltham, MA, USA). Each spectrum is the average of 32 sample scans between 400 and 4000 cm^−1^_,_ with a resolution of 4 cm^−1^. The FTIR 2D maps were recorded with a Nicolet iN10 MX (Nicolet, Waltham, MA, USA).

A JASCO (Jasco Inc., Easton, PA, USA) V560 spectrophotometer equipped with a 60 mm integrating sphere (ISV-469) was used to record the UV-Vis spectra. All the measurements were made in the domain 200–900 nm, with a speed of 200 nm min^−1^. The opacity values were calculated as A_600_/x = −logT_600_/x, where A_600_ is the absorbance at 600 nm, T_600_ is the fractional transmittance at 600 nm and x is the film thickness in mm [4].

A Perkin Elmer LS55 (Perkin Elmer, Waltham, MA, USA) fluorimeter was used to record the photoluminescence spectra (PL). A Xe lamp was used as an excitation source at ambient temperature. The excitation wavelength was 320 nm. The emission spectra were recorded in the domain 350–650 nm, with a scan speed of 200 nm min^−1^, a 350 nm cut-off filter, and slits of 10 nm.

Scanning electron micrographs for the determination of the films surface morphology and microstructure were obtained using a QUANTA INSPECT F50, FEI Company, Eindhoven, The Netherlands, scanning electron microscope equipped with a field emission gun—FEG with 1.2 nm resolution and an energy dispersive X-ray spectrometer (EDS) with an MnK resolution of 133 eV. Samples were coated with a thin gold layer to ensure conductivity.

Mechanical property analysis was performed using a 5965 universal testing machine (Instron, Norwood, MA, USA) with a 5 kN load cell and a crosshead speed of 50 mm/min, according to the EN ISO 527 standard [80]. The tested specimen was type 1B (10 ± 0.1 mm width at the narrow portion and 0.36 ± 0.06 mm thickness).

The permeability to water vapors was measured using a Lyssy L80-5000 tester (Dansensor, Ringsted, Denmark) with automatic temperature control and humidity sensor, according to EN ISO 15106-1 standard [81], while the permeability to oxygen was measured with a manometric tester, Lyssy L100-5000 (Dansensor, Ringsted, Denmark), according to ISO 2556:2000 [82]. For both permeability to water vapors and oxygen, samples with dimensions of about 13 × 13 mm were affixed to the self-adhesive sample cards provided with the equipment.

#### 3.1.3. Antimicrobial Assay

An antimicrobial assay was evaluated against model Gram-positive *Staphylococcus aureus* ATCC 25923 and Gram-negative *Escherichia coli* ATCC 25922 bacterial strains. To qualitatively screen the antibacterial effect of the obtained materials, we utilized an adapted diffusion assay, respecting the general rules exposed in the CLSI 2020 and in our recent study [5]. Bacterial suspensions (0.5 McFarland, 1.5 × 10^8^ CFU/mL) were prepared in sterile saline solution (0.9% NaCl) and were further utilized as a standardized inoculum to swab inoculate Petri dishes containing nutritive agar. Discs of 6 mm in diameter from the composite films were sterilized by UV exposure for 30 min before use. Sample discs were aseptically placed on the inoculated Petri dishes, and these were incubated for 20 h at 37 °C. After incubation, the diameter of growth inhibition developed around each material specimen was measured and expressed in mm.

The antibiofilm efficiency tests were performed by introducing sterile 6 mm-diameter disks, from each composite film into sterile 24-well plates containing 1 mL of nutritive broth, followed by the inoculation of 10 μL of bacterial suspension of 0.5 McFarland standard density. The as prepared plates were incubated for 24 h at 37 °C. After 24 h the disks were gently washed with 1 mL of sterile saline and transferred to centrifuge tubes containing 1 mL of sterile saline solution. The tubes were vortexed for 30 s to ensure the detachment of biofilm cells in suspension. Serial 10-fold dilutions were obtained and then inoculated on nutrient agar in order to evaluate the viable colony formation, expressed as CFU (colony-forming units)/mL. All experiments were designed and performed in triplicate.

### 3.2. Synthesis of ZnO Nanoparticles

ZnO synthesis was performed by forced solvolysis, as described previously in [83] with modifications from [32]. Shortly, 2.1950 g of Zn(CH_3_COO)_2_∙2H_2_O was mixed with 50 mL of n-butanol and heated to boiling point under vigorous magnetic stirring for 10 h. The resulting precipitate was washed twice with water and finally with ethanol. The separation was done by centrifugation, and the obtained powder was further dried at 105 °C.

### 3.3. Synthesis of Polyethylene/ZnO Composite Films

The mixtures (LDPE, LDPE-g-MA, glycerol, and ZnO NPs) in quantities from Table 7 were introduced in an internal Plasti-Corder Brabender mixer at 150 °C with a mixing rate of 50–70 rpm for 10 min.

**Figure 16:**
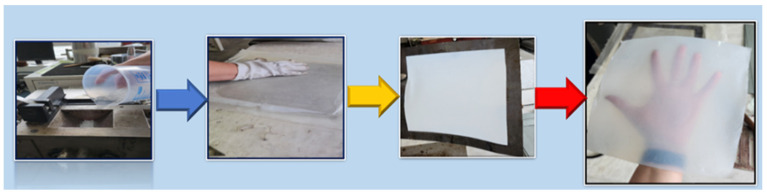
The schematic obtaining polyethylene-ZnO films.

The resulting mixtures were used to make polyethylene/ZnO composite films using specific moulds and the laboratory electrical press. The metal frame with dimensions of 245 × 300 mm was pressed between metal plates. The processing parameters were: preheating for 2 min at the temperature of 160 °C; modelling at the temperature of 160 °C; with a pressing force of 300 kN for 10 min; followed by the cooling down stage to 45 °C. The schematic process is depicted in Figure 16.

## 4. Conclusions

In this study, antibacterial packaging films were prepared by introducing ZnO NPs into the polyethylene matrix. The result indicates that even the low concentration of 1% ZnO is enough for the antibacterial and antibiofilm activity to kick in. Innovative antimicrobial packaging films based on polyethylene and ZnO nanoparticles were obtained in a simple melt mixing process at 150 °C for 10 min with the help of a Plasti-Corder Brabender mixer. The FTIR microscopy and SEM micrographs indicate that the ZnO nanoparticles were homogenously dispersed into the polyethylene matrix. The new composite PE-ZnO films exhibited higher thermal stability vs. the simple polyethylene film up to 400 °C. The light barrier properties of the new composite are very good in the UV domain, with a transmittance as low as ~4% at 361 nm, but with high transparency in the visible domain. This is especially important as the high energy photons can induce food alteration by interacting with lipids or proteins. The introduction of ZnO into the composite has not altered dramatically the transparency of the packaging films in the visible domain, allowing customers to see the packed food. Barrier properties are improved by introducing the ZnO NPs into the polyethylene matrix; the mechanical and barrier properties are optimum at a concentration of 3% ZnO. Therefore, improvements in the properties of novel polyethylene composite films will be beneficial for many applications, in particular the packaging of food products, which are very sensitive to oxygen and UV light.

## Figures and Tables

**Table 1:** A_600_ values, thickness (mm), and opacity for polyethylene and composite films.

Sample	PE	PE-g-MA	PE_ZnO 1%	PE_ZnO 3%	PE_ZnO 5%
Absorbance (A_600_ nm)	0.121	0.171	0.224	0.278	0.323
Thickness (mm)	0.31 ± 0.01	0.36 ± 0.06	0.29 ± 0.01	0.30 ± 0.02	0.27 ± 0.02
Opacity	0.39	0.47	0.77	0.92	1.19

**Table 2:** The main FTIR peaks for polyethylene/ZnO samples.

Group Frequency (cm^−1^)	Functional Group/Assignment
2914	methylene asymmetric stretch (from LDPE)
2848	methylene symmetric stretch (from LDPE)
1472	crystalline bending methylene (CH_2_)
1463	amorphous bending methylene (CH_2_)
729–730	crystalline rock methylene (CH_2_)
717–718	amorphous rock methylene (CH_2_)
484–488	Zn-O bond

**Table 3:** Principal data from thermal analysis of polyethylene/ZnO composite films.

Sample	T5% (°C)	T10% (°C)	T15% (°C)	Mass Loss (%) RT-300 °C	T_onset_ (°C) (melting)	Melting Peak (°C)	Mass loss (%) 300–400 °C	ΔH_melting_ (J/g)	Crystallinity Degree (%)
PE-g-MA	334.0	366.5	380.3	2.06%	108.6	117.0	24.87	88.08	30.06
PE_ZnO 1%	407.5	414.6	419.0	1.19%	106.1	115.5	2.69	87.93	30.01
PE_ZnO 3%	407.9	415.7	419.9	1.00%	106.8	117.4	2.01	80.61	27.51
PE_ZnO 5%	413.6	420.7	425.3	1.24%	106.8	114.3	2.68	81.73	27.89

**Table 4:** Mechanical and barrier properties for polyethylene/ZnO composite films.

Sample	PE-g-MA	PE_ZnO 1%	PE_ZnO 3%	PE_ZnO 5%
Thickness (mm)	0.36 ± 0.06	0.29 ± 0.01	0.30 ± 0.02	0.27 ± 0.02
Elongation at break (%)	32.5 ± 2.9	38.0 ± 1.6	46.1 ± 3.2	14.6 ± 1.5
Tensile strength (MPa)	15.2 ± 0.33	17.1 ± 0.33	18.9 ± 0.16	15.5 ± 0.16
O_2_ permeability (cm^3^/m^2^·bar·day)	65.00 ± 0.02	63.02 ± 0.01	49.56 ± 0.02	36.78 ± 0.02
H_2_O vapor permeability (g/m^2^·day)	0.280 ± 0.007	0.125 ± 0.005	0.091 ± 0.002	0.088 ± 0.002

**Table 5:** Diameter of the growth inhibition zone (mm) for polyethylene/ZnO composite films.

Sample/ Strain	PE_ZnO 1%	PE_ZnO 3%	PE_ZnO 5%
*S. aureus*	9 ± 1.0	10 ± 1.0	11 ± 1.0
*E. coli*	10 ± 1.0	10 ± 1.0	12 ± 1.0

**Table 6:** Weight loss for tomatoes coated with composite films during storage.

Sample	Weight Loss (%)			
	1 Day	3 Days	10 Days	14 Days
PE-g-MA	0.31 ± 0.05	0.96 ± 0.12	3.71 ± 0.16	6.10 ± 0.25
PE_ZnO 1%	0.11 ± 0.03	0.23 ± 0.07	0.91 ± 0.11	1.74 ± 0.16
PE_ZnO 3%	0.07 ± 0.02	0.16 ± 0.03	0.70 ± 0.11	1.46 ± 0.11
PE_ZnO 5%	0.03 ± 0.01	0.11 ± 0.03	0.31 ± 0.07	0.82 ± 0.06

Similar reports on strawberries [34], lettuce [48], meat [26], or seafood [13] were found in the literature.

**Table 7:** The polyethylene/ZnO film composition and labels.

Sample Code	LDPE (g)	LDPE-g-MA	ZnO NPs (g)	Glycerol (g)
PE	248	0	0	2
PE-g-MA	238	10	0	2
PE_ZnO 1%	235.5	10	2.5	2
PE_ZnO 3%	230.5	10	7.5	2
PE_ZnO 5%	225.5	10	12.5	2

## Data Availability

The data presented in this study are available upon request from the corresponding authors.

## Supplementary Materials

The following supporting information can be downloaded at: https://www.mdpi.com/article/10.3390/ijms25116073/s1.
