# Supplementary material for: Antimicrobial Packaging for Plum Tomatoes Based on ZnO Modified Low-Density Polyethylene
Source: Int J Mol Sci. 2024 May 31;25(11):6073. doi: 10.3390/ijms25116073 (PMC11172566; doi:10.3390/ijms25116073)
Supplement: Supplementary file 1 [file ijms-25-06073-s001.zip › ijms-2999525-supplementary.pdf]

International Journal of *Molecular Sciences*

MDPI

Article

Antimicrobial packaging for plum tomatoes based on ZnO modified low density polyethylene

Ludmila MOTELICA[1,2], Denisa FICAI[1,2], Ovidiu-Cristian OPREA[1,2*], Roxana-Doina TRUSCA[1], Anton FICAI[1,2], Maria Daniela STELESCU[3], Maria SONMEZ[3], Mihaela NITUICA[3], Gabriel MUSTATEA[4], Alina Maria HOLBAN[1,5]

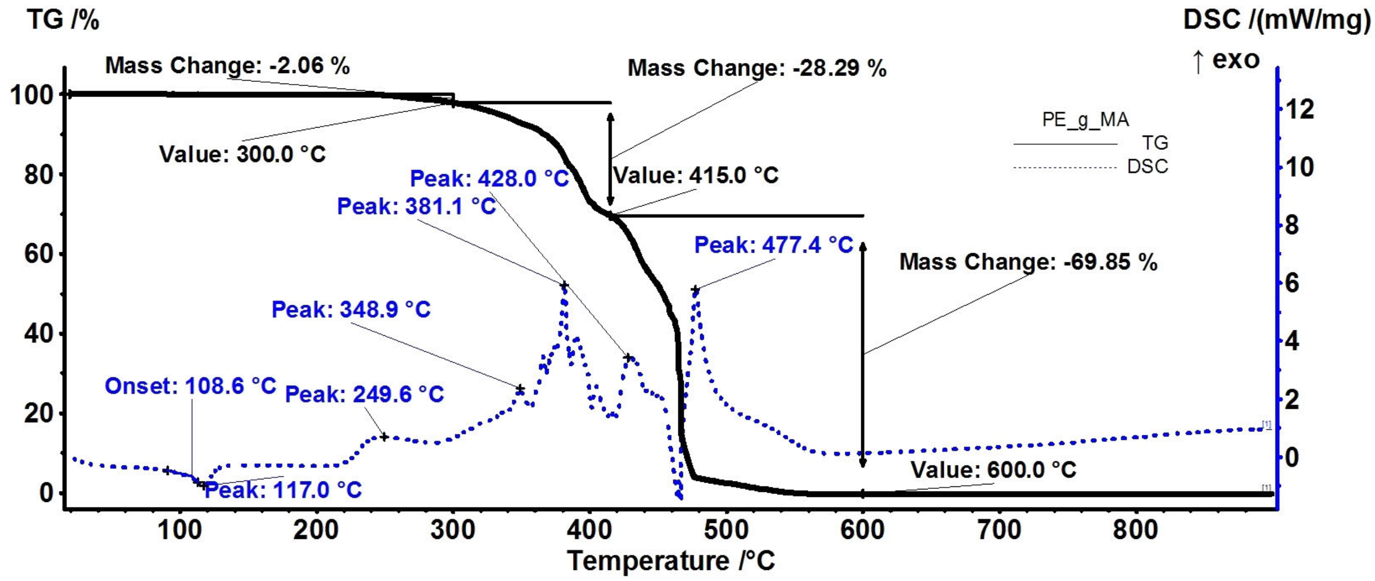

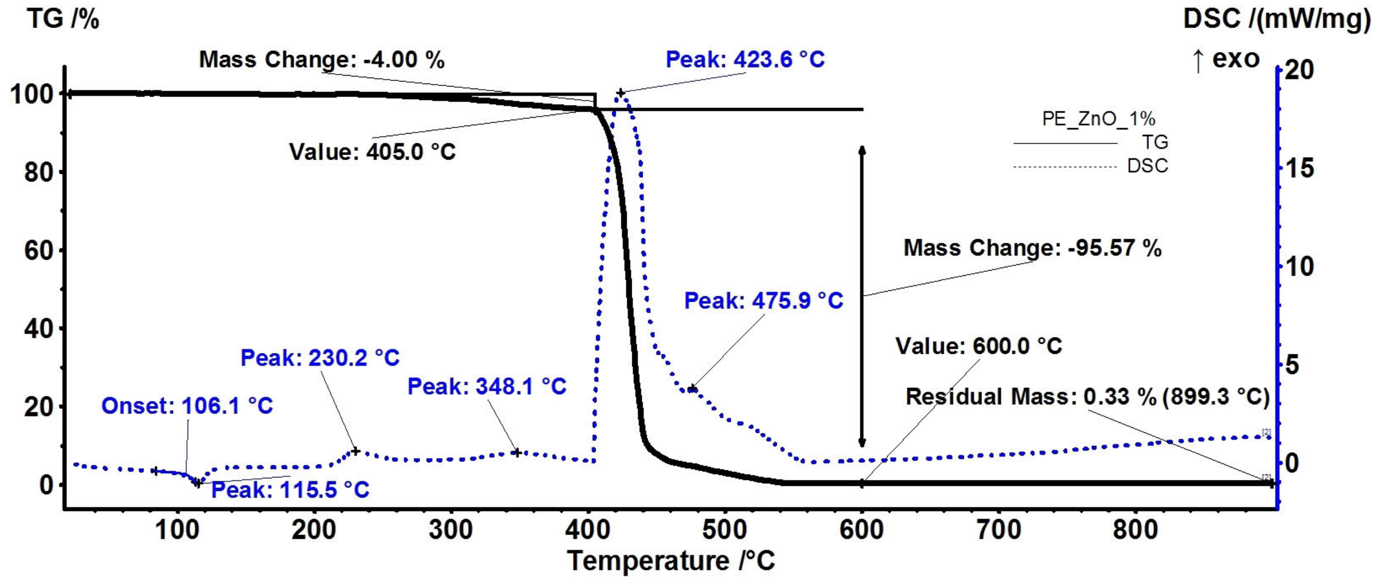

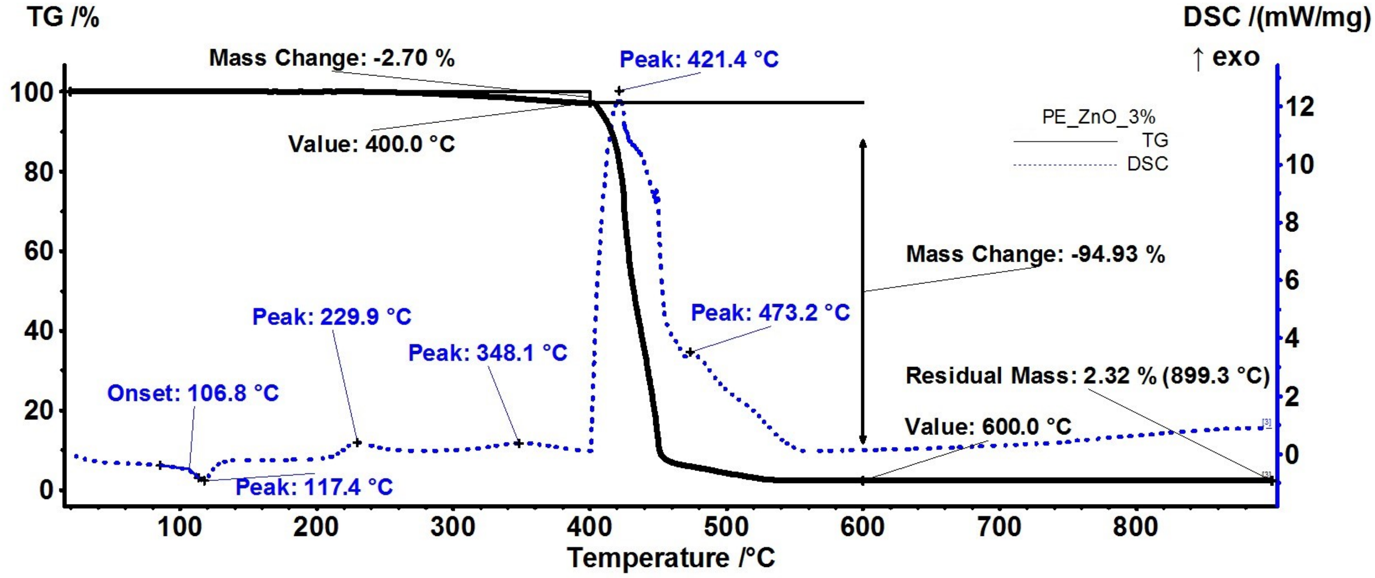

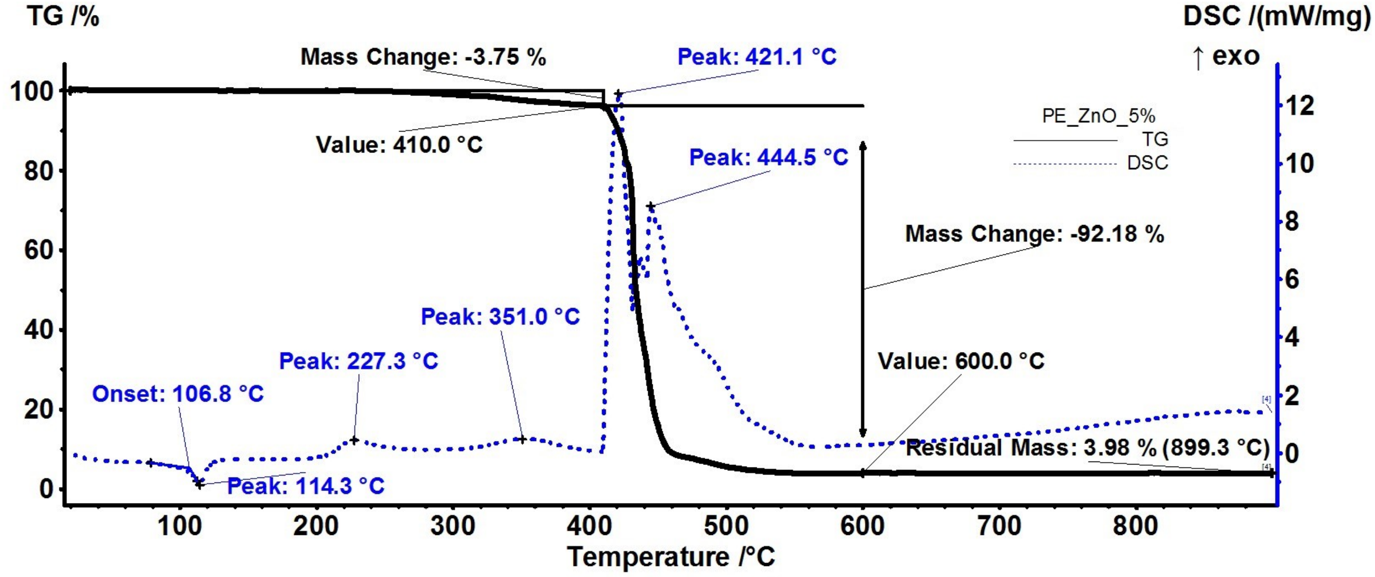

Figure S1. The numerical values of thermal analysis as measured on TG and DSC curves

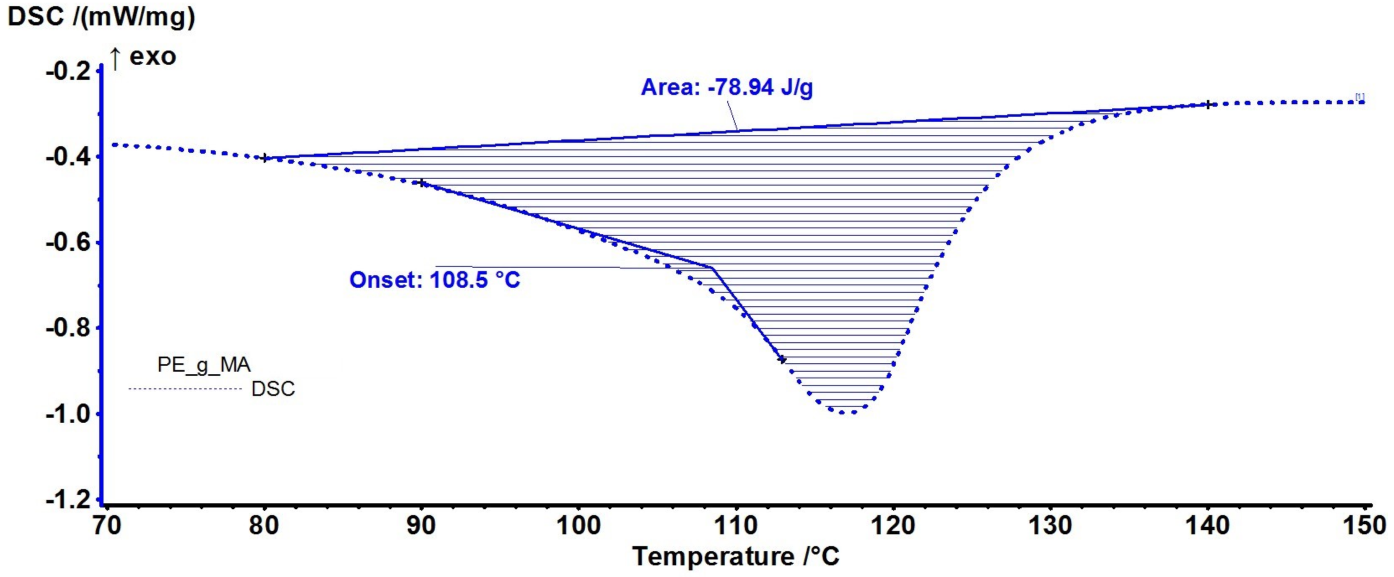
DSC /(mW/mg)
↑ exo
Area: -78.94 J/g
Onset: 108.5 °C
PE_g_MA
DSC
-0.2
-0.4
-0.6
-0.8
-1.0
-1.2
70
80
90
100
110
120
130
140
150
Temperature /°C

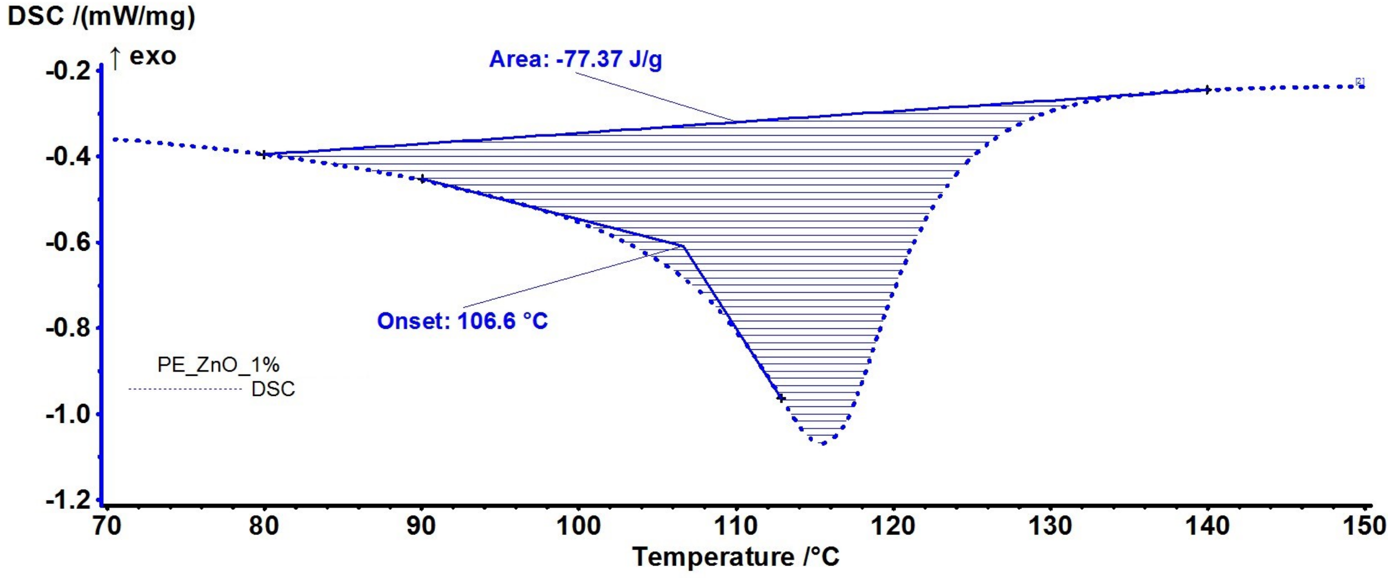
DSC /(mW/mg)
↑ exo
Area: -77.37 J/g
Onset: 106.6 °C
PE_ZnO_1%
DSC
-0.2
-0.4
-0.6
-0.8
-1.0
-1.2
70
80
90
100
110
120
130
140
150
Temperature /°C

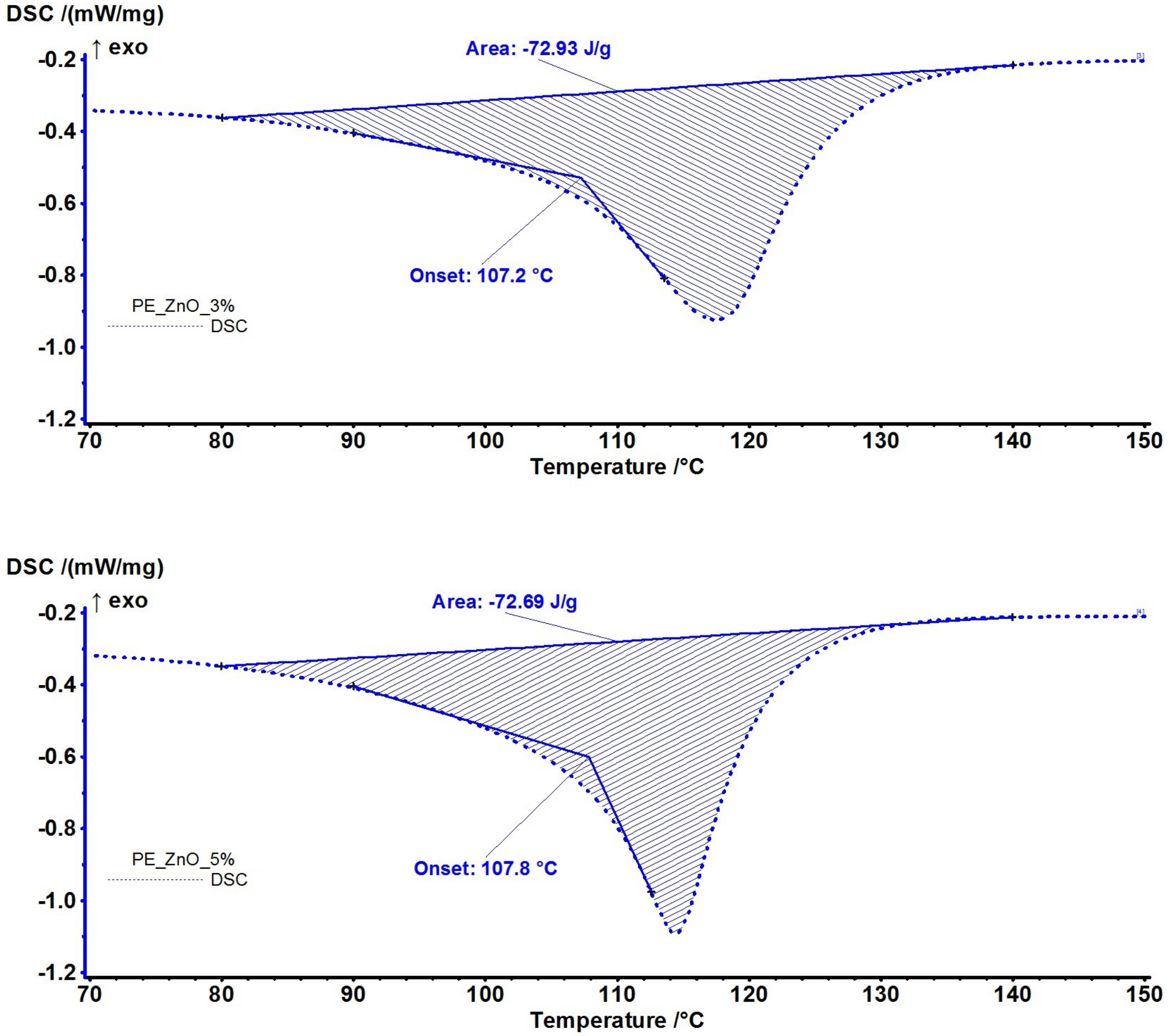

Figure S2. DSC curves and determination of melting enthalpy and melting onset.
